# The diagnostic value of prenatal ultrasound imaging features in an infant with congenital human cytomegalovirus infection combined with blueberry muffin-like skin manifestations: a case analysis and literature review

**DOI:** 10.1186/s12887-026-06991-4

**Published:** 2026-05-12

**Authors:** Wenjing Gao, Wei Shi, Qi Lin, Weiyue Li, Shaofu Hong, Qihui Peng, Fajin Dong, Xu Zhang

**Affiliations:** 1 Department of Ultrasound, Shenzhen People’s Hospital (The First Affiliated Hospital, Southern University of Science and Technology; The Second Clinical Medical College, Jinan University), Shenzhen, 518020 China; 2 Department of Obstetrics, Shenzhen People’s Hospital (The First Affiliated Hospital, Southern University of Sicence and Technology; The Second Clinical Medical College, Jinan University), Shenzhen, 518020 China; 3 Department of Radiology, Shenzhen People’s Hospital (The First Affiliated Hospital, Southern University of Sicence and Technology; The Second Clinical Medical College, Jinan University), Shenzhen, 518020 Guangdong China; 4 Department of Ultrasound, the Second People’s Hospital of Shenzhen (the First Affiliated Hospital of Shenzhen University), Shenzhen, 518020 Guangdong China

**Keywords:** Blueberry muffin rash, Prenatal ultrasound, Congenital cytomegalovirus, Diagnostic analysis

## Abstract

**Background:**

Congenital cytomegalovirus (CMV) is the most common congenital viral infection worldwide. Most infants with CMV have no obvious clinical symptoms at birth. Purplish–blue skin ecchymosis occurs in a small number of symptomatic neonates with CMV, which is rare.

**Case presentation:**

A 36-year-old woman experienced menses cessation for 37 weeks and 6 days. The foetal ultrasound diagnostic assessment suggested the following: (1) Bilateral subependymal cysts and mild ventriculomegaly involving the lateral and third ventricles. (2) Doppler assessment and foetal biometric measurements suggested the possibility of moderate foetal anaemia and foetal growth restriction (FGR). CMV infection was ruled out because of concurrent subependymal cysts and anaemia. The pregnant woman underwent emergency caesarean section for foetal distress and delivered a live male infant with a birth weight of 1.97 kg. The newborn presented with a generalized purplish–black rash (predominantly facial), thrombocytopenia, bilateral hearing screening failure (confirmed by AABR), and right retinal haemorrhage. Postdelivery, maternal serology revealed elevated cytomegalovirus IgG antibodies (1436.6 AU/mL). The final diagnoses included neonatal CMV infection and blueberry muffin rash.

**Conclusions:**

Neonatal blueberry muffin rash caused by CMV infection is rare in the clinic. This rare case demonstrates the certain role of prenatal ultrasound in detecting CMV-associated anomalies and highlights the importance of multidisciplinary correlations (clinical, laboratory, and imaging findings) for the timely diagnosis of neonatal blueberry muffin rash.

**Supplementary Information:**

The online version contains supplementary material available at 10.1186/s12887-026-06991-4.

## Background

Congenital cytomegalovirus (CMV) infection is recognized as the most prevalent congenital viral infection worldwide. CMV infection is also an important cause of hearing loss and neurological disability in children [1, 2], affecting approximately 0.2%–6.1% of live births worldwide [3, 4]. Most infants with CMV have no obvious clinical symptoms at birth, and fewer than 10% of newborns with CMV have multiple clinical manifestations (symptomatic CMV). CNV can manifest as intrauterine growth restriction, a cutaneous purplish colour after birth, hepatitis, chorioretinitis, neurogenic deafness, etc. Infants with central nervous system (CNS) involvement also have microcephaly, neuroimaging abnormalities (ventriculomegaly, intracerebral calcification, white matter changes), and seizures [5]. Laboratory tests can reveal thrombocytopenia, elevated aminotransferase, bilirubin, CMV IgM antibody, and IgG antibody levels, and CMV nucleic acid positivity. Cutaneous purplish–blue ecchymosis is rare in symptomatic neonates with CMV (incidence < 5%) and does not fade under pressure. This condition is known as blueberry muffin rash because of its resemblance to a blueberry muffin. The term “blueberry muffin rash” was first used to describe purpura skin damage caused by extramedullary haematopoiesis in newborns born with congenital rubella infection during the American rubella epidemic of the 1960s. There are many extramedullary haematopoietic organs involved in the normal development of embryos. Infants with congenital blueberry muffin rash have haematopoietic organs, causing abnormal rashes, and the histopathological manifestation is dermal erythropoiesis [6]. “The blueberry muffin rash” is mainly characterized by the appearance of blue-red rashes and papules all over the body. The blue‒red rash, which does not fade under pressure, occurs mostly on the trunk, head and neck, begins to subside after birth and disappears completely after 3–6 months. Diseases that can cause blueberry muffin rash include congenital infections, including TORCH syndrome (toxoplasma, rubella, cytomegalovirus, herpesvirus, and others), disorders of the blood system, metabolic disorders, neoplastic diseases, and congenital vascular diseases [7, 8]. Among congenital infections associated with TORCH syndrome, CMV infection is the most common, occurring in 0.3–2.2% of infants. Approximately 40% to 60% of symptomatic newborns with CMV have permanent sequelae, the most common of which is sensorineural hearing loss (SNHL), followed by cognitive impairment, chorioretinitis, seizures, and even death [9]. Informal International Congenital Cytomegalovirus Recommendations recommend the use of valaciclovir for 6 months for symptomatic newborns with moderate-to=severe congenital CMV (multiple manifestations or CNS involvement). However, antiviral therapy is not recommended for mildly symptomatic patients with congenital CMV and one or two isolated manifestations or asymptomatic patients [1].

Although CMV is the most common viral infection worldwide, the diagnosis of CMV is still difficult because routine CMV screening is not recommended for all pregnant women [10, 11]. Foetal ultrasound plays a pivotal role throughout pregnancy, with the advantages of noninvasiveness, no radiation exposure, repeatability and dynamic observation. Ultrasound can be used to periodically assess foetal size and development, screen for most congenital malformations, and evaluate foetal circulation and foetal appendages. Foetal ultrasound plays an important role in the diagnosis of CMV, and the positive predictive value of CMV is nearly 50% [12]. The most common ultrasonic manifestations of CMV include cerebral calcification (0.6%–17.4%), microcephaly (14.5%), a strong intestinal echo (4.5%-13%), foetal growth restriction (FGR) (1.9%–13%), ependymal cysts (11.6%), ascites (8.7%), pericardial effusion (7.2%), an enhanced renal echo (4.3%), hepatomegaly (4.3%), etc [10, 11]. Abnormal manifestations on foetal ultrasound are highly important for the screening and prenatal diagnosis of congenital CMV. In addition, neuroimaging abnormalities of CMV can manifest as ventriculomegaly, intracerebral calcification, and white matter changes.

## Case presentation

A 36-year-old pregnant woman who experienced period cessation for 37 weeks and 6 days was found to have a foetus that was two months smaller than expected at another hospital. She was subsequently admitted to our hospital for further diagnosis and treatment. The pregnant woman had no obvious discomfort in the first or second trimester. She had no history of vaginal bleeding, lower abdominal pain, cold or fever, radiation exposure, exposure to chemical poisons, etc. No significant abnormalities were detected by prenatal examination or foetal ultrasound during early or middle pregnancy. The detailed results of the foetal ultrasound performed at 29 + weeks of gestation at the other hospital were unknown. According to foetal ultrasound, the biparietal diameter (BPD) of the foetus was 66 mm, the femoral length (FL) was 49 mm, and the size of the foetus was estimated to be approximately 26 + weeks of gestation. An oral glucose tolerance test (OGTT) performed at 29 + weeks of gestation revealed fasting/1-hour/2-hour glucose levels of 5.21, 10.19, and 8.31 mmol/L, respectively, confirming the diagnosis of gestational diabetes mellitus. No special treatment or follow-up was provided.

**Fig. 1:**
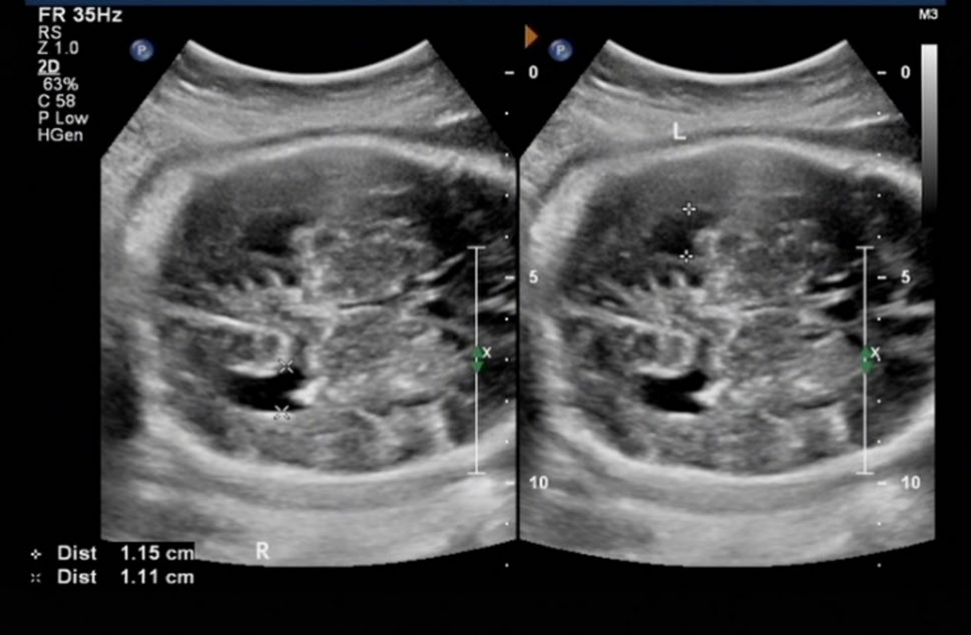
The bilateral lateral ventricles are enlarged: 11.1 mm (right) and 11.5 mm (left)

**Fig. 2:**
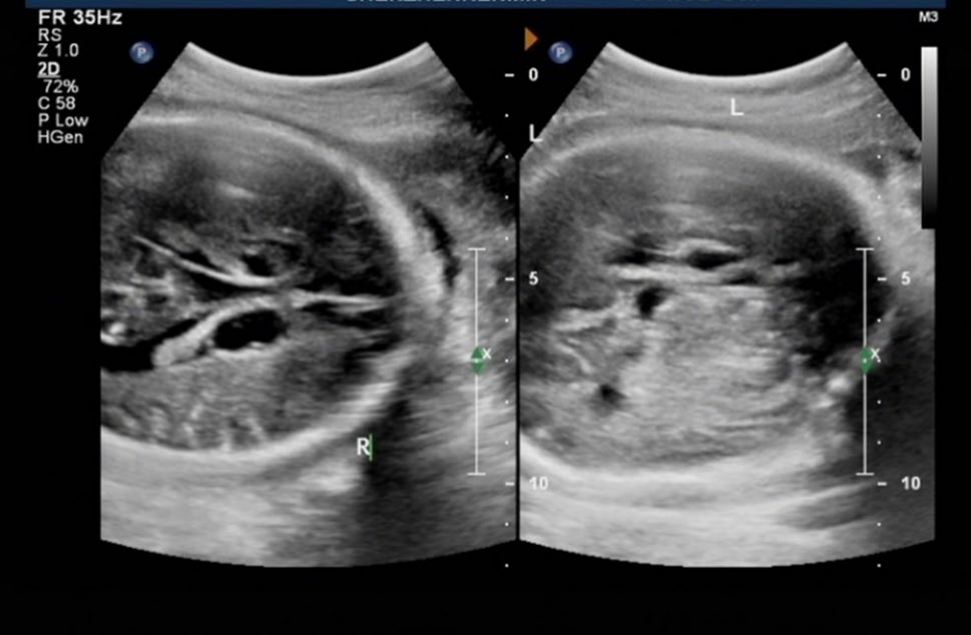
Anechoic areas at the anterior horns of the lateral ventricles: 17 × 10 mm (right) and 16 × 7 mm (left)

**Fig. 3:**
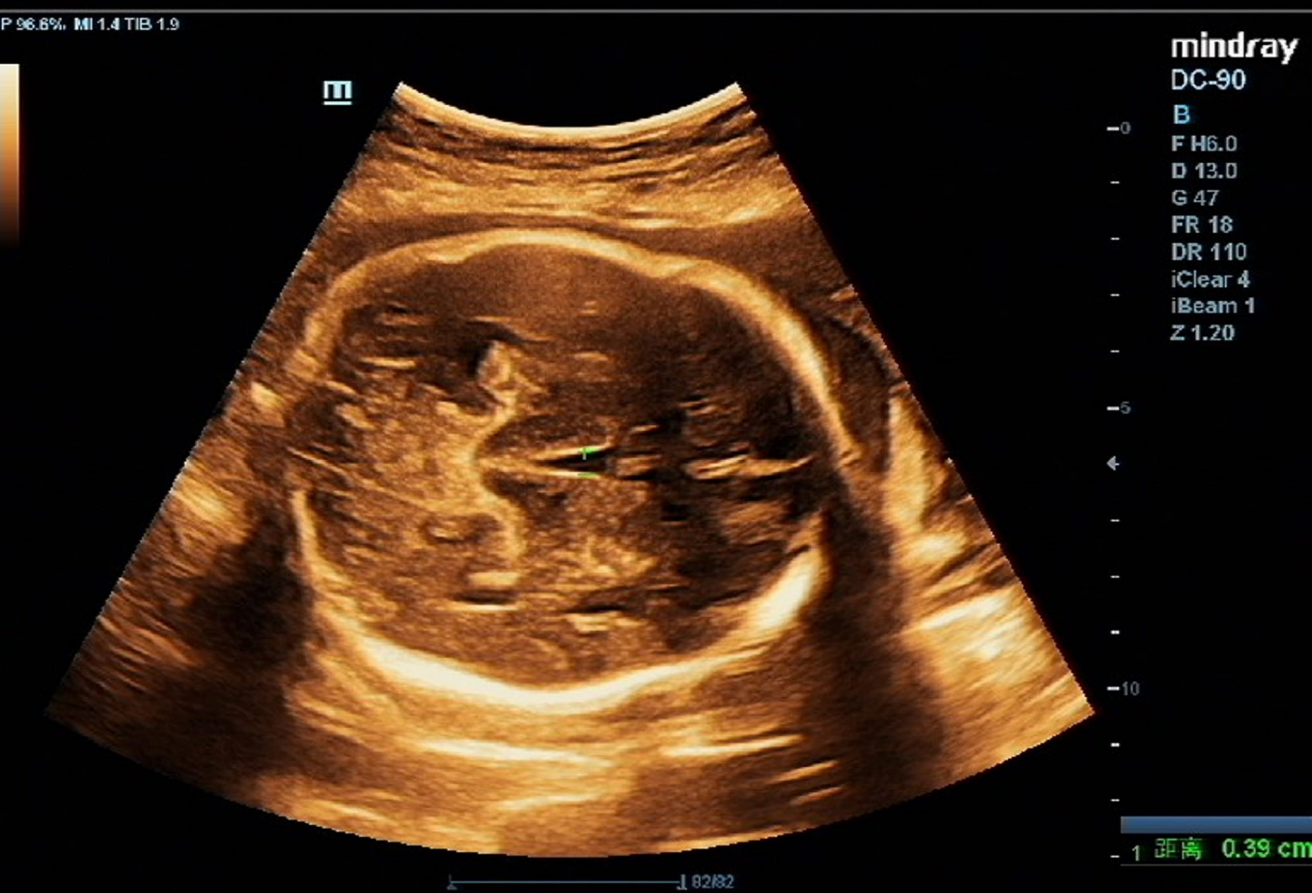
The widened third ventricle:3.9 mm

Foetal ultrasound examination was performed immediately after admission. The grayscale ultrasound findings were as follows. The bilateral lateral ventricles demonstrated mild dilation, with a left ventricular width of approximately 11.5 mm and a right ventricular width of 11.1 mm (Fig. 1). Anechoic areas were observed at the lateral edges of the anterior horns of both lateral ventricles, measuring approximately 16 × 7 mm (left) and 17 × 10 mm (right) (Fig. 2). The third ventricle was slightly widened, measuring approximately 3.9 mm in width (Fig. 3). The corpus callosum, midline structures, lateral ventricles, and thalami were visualized, with no apparent structural defects. The placenta and amniotic fluid volume appeared within normal limits.

**Fig. 4:**
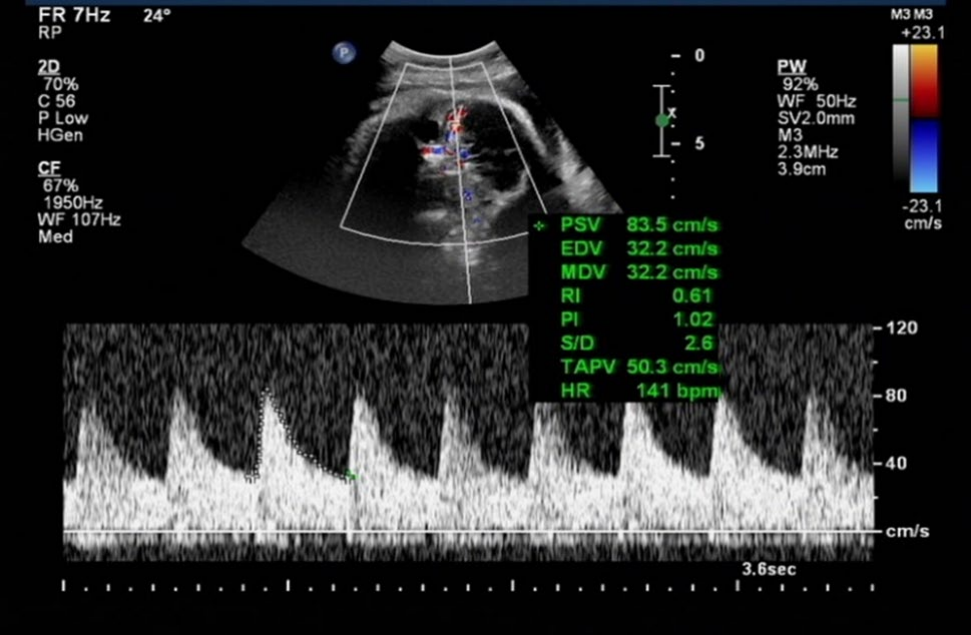
The peak systolic velocity of the MCA (Fig. 4, 84 cm/s, 1.51 MoM)

**Fig. 5:**
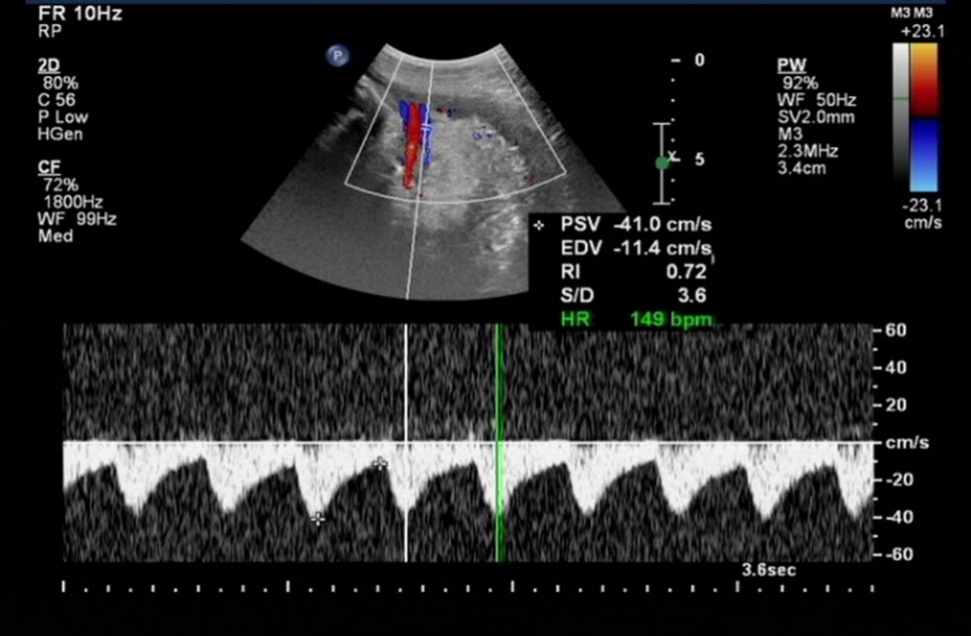
The umbilical artery resistance index (Fig. 5, S/D: 3.6, PI: 1.02) were increased

**Fig. 6:**
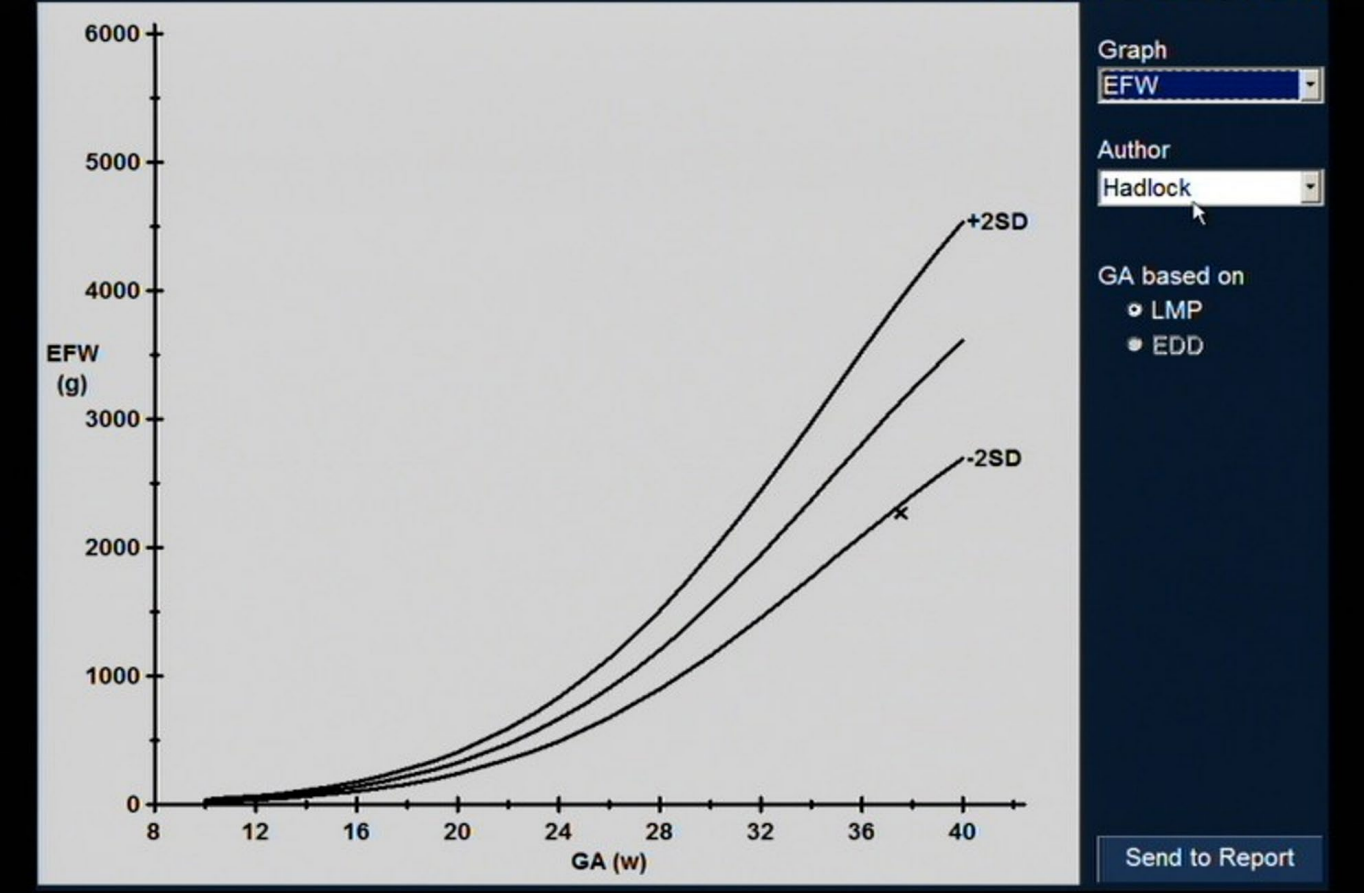
The EFW was 2 SDs lower than the average for other infants of the same gestational age

Doppler assessment revealed that the peak systolic velocity of the middle cerebral artery (MCA: 84 cm/s, 1.51 MoM) and the umbilical artery resistance index (S/D: 3.6, PI: 1.02) were increased (Figs. 4 and 5). The foetal biometric measurements were as follows: (1) BPD: 83 mm (equivalent to 33 + weeks gestation); (2) head circumference (HC): 305 mm (33 + weeks); (3) abdominal circumference (AC): 306 mm (34 + weeks); (4) FL: 63 mm (32 + weeks); and (5) humerus length (HL): 56 mm (32 + weeks). The estimated foetal weight (EFW: 2270 g) was 2 SDs lower than the average for other infants of the same gestational age (Fig. 6).

In conclusion, the foetal ultrasound findings suggested the following: (1) Bilateral subependymal cysts and mild ventriculomegaly involving the lateral and third ventricles. (2) Doppler assessment and foetal biometric measurements suggested the possibility of moderate foetal anaemia and FGR.

Placental insufficiency was less likely given the normal placental and amniotic fluid indices, and CMV infection was ruled out because of concurrent subependymal cysts and anaemia. Further prenatal counselling (e.g., foetal MRI, CMV serology, and cordocentesis) was recommended.

**Fig. 7:**
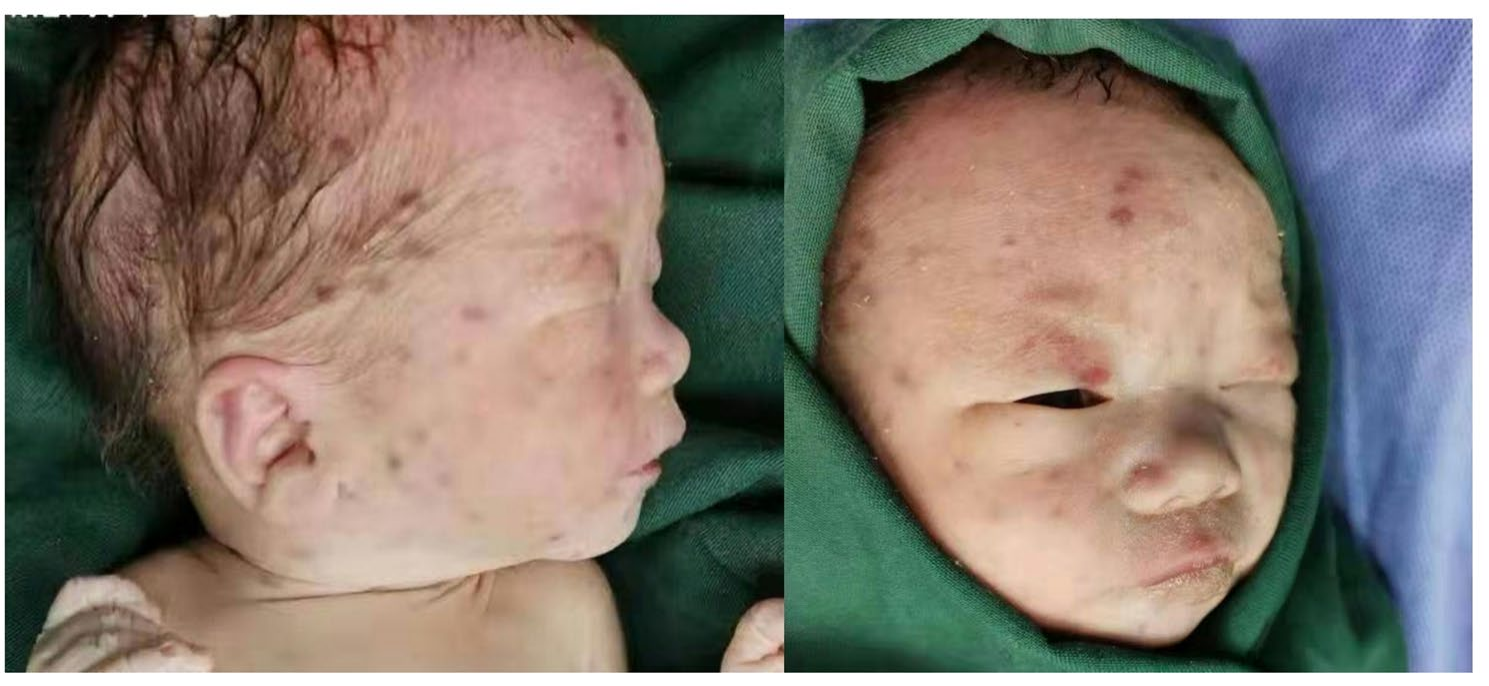
A purplish-black rash can be seen on the whole body of the newborn, especially on the face

Moreover, obstetrical foetal heart monitoring revealed repeated late deceleration, which suggested the possibility of foetal distress. The patient underwent a caesarean section under general anaesthesia immediately at our hospital due to “foetal distress” and delivered a live baby. The Apgar score at 1, 5 and 10 min after birth was 8 points (1 point for breath and 1 point for skin colour), 9 points (1 point for skin colour) and 10 points, respectively. The newborn’s body weight was obviously low: 1.97 kg. A purplish–black rash was observed on the newborn’s entire body, especially on the face, which was not greater than the skin surface and did not fade under pressure (Fig. 7). The preliminary clinical diagnosis was CMV infection. The newborn was then transferred to the neonatology department with the consent of the family. Laboratory examination of the newborn revealed thrombocytopenia at birth (29.00 × 109/L), and the lowest value was 9.00 × 109/L on the 6th day after delivery. TORCH revealed a cytomegalovirus IgM antibody concentration of 1.9 S/CO, a cytomegalovirus IgG antibody concentration of 2059.4 AU/ml, a urine CMV nucleic acid concentration of 3.70E6 copies/ml, and a blood cytomegalovirus (CMV) nucleic acid concentration of 8.86E2 copies/ml. The newborn failed the hearing screening and AABR for both ears. Fundus screening revealed flaky bleeding in the right retina. Head MRI revealed the following: 1. dilation of the bilateral ventricles and bilateral subependymal cysts and calcification of the right basal ganglia and left ventricle, possibly indicating TORCH syndrome. Cerebrospinal fluid examination revealed no significant abnormalities. Laboratory tests performed after the emergency caesarean section revealed an elevated concentration of IgG antibodies against cytomegalovirus in the woman: 1436.6 AU/ml. The final diagnoses for the newborn were neonatal CMV infection and blueberry muffin rash. After 4 weeks of symptomatic treatment with anti-infectives, platelet transfusions, hormones and globulin, the newborn’s platelet count increased to 88.00 109/L, and the rash partially subsided. The newborn’s family refused further treatment, and the newborn and was discharged. The infant was followed up by telephone at 8 months and 1 year after delivery and had still not passed the hearing examination. Neurobehavioral examination was not performed, and cochlear implantation was performed at a later date. During the phone follow-up when the child was four years old, the child could hear and speak simple words during active rehabilitation language training, and there was no obvious intellectual development abnormality.

## Discussion and conclusion

Blueberry muffin baby is a descriptive term for purpuric lesions reflective of extramedullary hematopoiesis. The clinical lesions most commonly result from intrauterine infections, and less commonly with malignancy and hematologic disorders [13]. This typical case of neonatal blueberry muffin rash caused by cytomegalovirus infection has relatively complete medical history and clinical data, which can provide reference and clinical experience for the diagnosis of such diseases in the future. The data includes prenatal ultrasound, maternal and infant laboratory tests, imaging examinations, postnatal screening of the newborn, and imaging examinations. Reviewing this case, during the foetal ultrasound screening at 29 weeks of pregnancy at another hospital, the foetus was found to be smaller than normal, estimated to be approximately 26 + weeks of gestation. However, the pregnant woman did not conduct further relevant examinations. It was not until 37 + weeks that she visited our hospital. After admission, foetal ultrasound examination was immediately conducted, revealing moderate foetal anaemia and the possibility of foetal growth restriction. Foetal growth restriction is mainly caused by placental dysfunction. It also may be due to maternal insults or pregnancy compromise including infection/inflammation, pregnancy diseases, eating disorders, and hormonal imbalance [14]. In this case, the growth restriction can be ruled out as a placental factor first, because the foetal ultrasound shows no abnormalities in the placenta. Furthermore, ultrasound also detected bilateral subependymal cysts in the foetus, with a sensitivity of 88% for congenital infections or genetic disorders [15]. Therefore, the main consideration is the possibility of maternal infection or foetal developmental abnormalities. The clinical recommendation is to perform amniocentesis and maternal laboratory tests to rule out infection. However, since the foetus may have intrauterine distress, an emergency caesarean section is recommended. Postnatally, the neonate’s low birth weight combined with the extensive purple‒black maculopapular rash (characteristic blueberry muffin lesions) strongly indicated haematologic involvement. Laboratory confirmation through CMV IgM/IgG seropositivity (1:5120 titre) and thrombocytopenia (12 × 10⁹/L) screening established a definitive diagnosis, aligning with established diagnostic criteria for congenital CMV syndrome. Moreover, the failure of both AABR and binaural hearing screening examinations, patchy retinal haemorrhage in the right eye, and abnormal brain MRI findings further confirmed the diagnosis of CMV infection.

After reviewing the literature, reports on the prenatal ultrasound characteristics of blueberry muffin infants caused by congenital cytomegalovirus infection are rare. Sofia [7] reported a case of a full-term pregnancy with cytomegalovirus infection in a blueberry muffin infant. The prenatal ultrasound diagnosis was intrauterine growth restriction of the foetus, which was consistent with ours. Furthermore, the ultrasound findings of the foetus in this case indicated foetal growth restriction and bilateral choroid plexus cysts, which are the common manifestations of CMV. This case demonstrates that prenatal ultrasound plays a crucial role in diagnosing cytomegalovirus infection, which is consistent with Guerra’s conclusion [9].

This case also has some shortcomings. (1) The detailed ultrasound screening results of the foetus during the early and middle stages of the pregnant woman’s pregnancy could not be traced. (2) During the follow-up of newborns, there is a lack of detailed records of the infants’ physical measurements, such as height and weight. In similar cases in future, a more comprehensive review of the medical history and a more detailed recording of the follow-up information should be conducted.

In conclusion, neonatal blueberry muffin rash caused by CMV infection is rare in the clinic. This rare case demonstrates the certain role of prenatal ultrasound in detecting CMV-associated anomalies and highlights the importance of multidisciplinary correlations (clinical, laboratory, and imaging findings) for the timely diagnosis of neonatal blueberry muffin rash. Through a review of the complete diagnosis and treatment process of this rare and typical case and a comprehensive analysis, we re-reviewed the relevant content about neonatal blueberry muffin rash. This has enhanced our understanding of this rare disease and will be of great help for its diagnosis in the future.

## Supplementary Information

Supplementary Material 1.

## Data Availability

No datasets were generated or analysed during the current study.
